# How can the collaborative participation of regulators, whistleblowers, and parties effectively promote rumor management in public health emergencies?

**DOI:** 10.3389/fpubh.2023.1290841

**Published:** 2024-01-08

**Authors:** Yalin Wang, Liping Qi, Shaoshuo Cai

**Affiliations:** ^1^Guangzhou Huashang College, Guangzhou, China; ^2^School of Humanities, University of Chinese Academy of Sciences, Beijing, China; ^3^School of Journalism and Communication, Hunan Normal University, Changsha, China

**Keywords:** public health emergencies, rumor management, collaborative governance, social sustainability, evolutionary games

## Abstract

To effectively address the mental health risks associated with public health emergencies, it is crucial to actively manage rumors. This study explores the dynamic evolutionary process of rumor diffusion and its collaborative governance in public health emergencies. A game-theoretic model is constructed, involving three main actors: regulators, parties involved in public health emergencies (PIPHE), and whistle-blowers. The behaviors and game outcomes of each party are analyzed, and the effectiveness and feasibility of the model are validated through numerical simulations. The findings of this study reveal that various factors, such as regulatory costs, penalty income, reputation damage for regulators; image loss, reputation enhancement, penalty expenditure for PIPHE; and time costs, social responsibility, and reward income for whistle-blowers, all influence the behavioral choices and game equilibrium of each party. Optimization strategies for rumor governance are proposed in this study, including enhancing the sense of responsibility and capability among regulators, increasing transparency and credibility among PIPHE, and encouraging and protecting the participation of whistle-blowers. This study provides a comprehensive analytical framework for rumor governance in public health emergencies, contributing to improving the governance of public health emergencies and maintaining online public health orders for social sustainability.

## 1 Introduction

In 2020, the emergence of a novel coronavirus (COVID-19) and its rapid global dissemination posed a significant test to the worldwide public health infrastructure. Similarly, a spectrum of public health incidents, ranging in similarities to COVID-19, such as climate change, health disparities, digital health, food safety, and mental health, due to their abrupt, uncertain, and hazardous nature, frequently engender public discourse and societal risks (1), particularly in the emergence and dissemination of rumors. The escalating ubiquity of social media platforms serves as a principal conduit for the dissemination of rumors, thereby amplifying the impact of such misinformation and instigating heightened and more frequent instances of societal discourse crisis (2). An illustrative example is the COVID-19 pandemic in 2020, wherein the initial rumor proposing that the Chinese medicine “Shuanghuanglian” could inhibit the novel coronavirus triggered a significant increase in purchases among Chinese citizens (3). These rumors further intensified social instability stemming from a crisis event.

In recent years, researchers have conducted numerous studies on how to manage rumors (4, 5). Rothkopf (6) first proposed the concept of “information epidemics,” arguing that rumors can affect a country’s economy, politics and national security, and ultimately the whole world. In circumstances marked by a dearth of authoritative information, rumors can cause more serious damage and credit crisis, thereby potentially culminating in disarray and augmenting the complexity of conflict resolution (7). As unverified information is iteratively presented and propagated, initially dubious rumors may progressively gain credibility during dissemination, leading to an amplification of risks and rendering individuals susceptible to the sway of collective emotions. This phenomenon exacerbates conflicts within the context of public health incidents (8), with panic-mongering rumors being the most socially damaging (9). Therefore, the governance of rumors is of great concern in all countries and regions and is an important area of social governance.

In current practice, rumor management of public health emergencies mainly starts with regulators and media, using traditional means such as deleting posts, dispelling rumors and media guidance (8). And the public often tends to search for more information to reduce uncertainty in a chaotic environment, which leads to the great spread of rumors (10, 11). Numerous studies have indicated the pronounced significance of stakeholder-oriented governance concerning rumors within the domain of public health incidents (12). Regulatory oversight, notably characterized by judicious legal and regulatory frameworks as well as administrative supervisory measures, emerges as a pivotal means for effectively preventing and dismantling the propagation of misinformation (2, 13).

In the process of rumor spreading and dissemination, parties involved in public health emergencies (PIPHE) play a crucial role (14), they have the responsibility to provide accurate and reliable information to dispel the rumors, and their timely response plays an important role in helping the regulatory authorities to prevent and intervene in large-scale rumor spreading (15). However, the reality is that many parties do not have this motivation and they may hide the facts for their own unilateral and short-sighted interests. In contrast, individuals and elites (whistle-blowers), emerge as the principal forces in countering rumors (16), adeptly accessing public health emergencies through social channels. Anchored upon evidence-based debunking strategies, their interventions exhibit considerable persuasiveness, and their oversight effectively contributes to the governance of rumors surrounding public health emergencies.

Risk communication strategies and health promotion among government, community, media, and patients, such as those adopted in the late stages of the Ebola epidemic in Africa, play an important role in preventing and responding to public health emergencies (17). The process also involves the participation of stakeholders such as social organizations, the public and the media (15, 18, 19). While an increasing body of research focuses on the governance of public health event rumors based on multi-agent dynamic analysis, significant variations persist in the study of key factors. Consequently, there is a lack of a comprehensive framework to analyze the spread of rumors during sudden public health events and formulate collaborative governance efforts between regulatory authorities and society. Nevertheless, such an integrated framework is of paramount importance for advancing the governance of rumors surrounding public health incidents.

According to our investigation of COVID-19-related health rumors, the very core stakeholders in the spread of online rumors during public health emergencies include regulators, PIPHE, whistle-blowers and the public. The attitudes and behaviors of regulators, parties, and whistle-blowers exert a significant influence on the public. To narrow the scope of inquiry, we designate the public as an exogenous participatory entity, with particular emphasis on the strategic interactions among the triadic entities: regulators, PIPHE, and whistle-blowers. This study centers on the dissemination of rumors regarding public health emergencies on social media and their collaborative governance.

Evolutionary game theory effectively describes a wide range of complex strategic interactions and decision-making processes in the real world (20, 21). Constructing mathematical models, allows for the formal analysis of different strategies and their interactions, aiding our understanding and explanation of behavioral phenomena in human society. The extensive application of this theory in various domains, such as industrial policy (22, 23), technology policy (21, 24), and environmental policy (25, 26), has provided valuable insights and inspiration to my work. Leveraging the framework of evolutionary game theory, we construct a dynamic game model encompassing regulators, PIPHE, and whistle-blowers. Within this construct, we analyze the behavioral strategies of each party in the context of rumor propagation, exploring gaming results. Furthermore, the model’s efficacy and feasibility are substantiated through numerical simulations. Subsequently, we delve into an exploration of the influential factors underpinning rumor dissemination, and we propose optimization strategies for collaborative governance involving regulatory entities and media outlets.

The primary objective of this study is to delve into the intricate dynamics of rumor spreading and dissemination, with a particular emphasis on the pivotal roles and interactions of key stakeholders during public health emergencies. The ultimate goal is to construct a framework based on evolutionary game that enables the thorough analysis of rumor propagation and facilitates the formulation of effective collaborative governance strategies between regulatory authorities and society. The innovations of this paper are (1) Adopting an evolutionary game-theoretic perspective, this study elucidates the intrinsic mechanisms underpinning the dissemination of rumors within the domain of public health emergencies, accounting for the rational choices and adaptive learning of all parties involved, as well as acknowledging the temporal dynamics and inherent uncertainty characterizing the propagation of rumors. (2) From the perspectives of regulators, PIPHE, and whistle-blowers, we analyze the process and results of the game of rumor propagation, as well as the interests and influence of each party, which provides the basis for the development of effective governance strategies. Particularly the analysis of whistle-blowers’ participation in rumor governance makes up for the shortcomings of existing studies.

## 2 Model design

### 2.1 Description of the problem

During public health emergencies, the spread of online rumors on social media poses a threat to citizens’ emotions and social stability, becoming a significant challenge in the field of public health (27). The regulation of rumors poses a systemic challenge that requires collaboration among regulators, PIPHE, and whistleblowers. The primary goal of regulators is to safeguard the public interest, which includes ensuring access to reliable information and maintaining social stability. Achieving this objective necessitates taking active measures to prevent the spread of misinformation and promptly disclosing relevant facts (28, 29). PIPHE may seek to gain public support and trust or protect their own interests. They should openly disclose information related to the events to alleviate public concerns and distrust (30). Whistleblowers may aim to expose the truth, assist regulatory agencies in detecting problems in a timely manner, and promote the implementation of effective governance measures. They can actively participate in controlling the proliferation of rumors (31).

However, both relevant literature (32, 33) and the facts we have investigated suggest that due to cost and benefit considerations, different stakeholders may not always adopt optimal strategies in a given situation. For example, in the early stages of the COVID-19 outbreak, there were instances of passive regulatory behavior among local authorities, PIPHE, and whistleblowers. However, as the situation developed, people became increasingly aware of the severity of the pandemic and adjusted their strategies accordingly. Moreover, in public health emergencies, there are complex interrelationships and interactions among these stakeholders. Regulators may face political pressure or be swayed by public opinion and opt for passive regulatory approaches. PIPHE stakeholders may use rumors to divert attention or enhance their reputation, leading them to conceal or delay information disclosure in an attempt to protect their standing. Whistleblowers may face legal risks or social ostracism and decide to remain silent or retract their claims.

**Figure 1:**
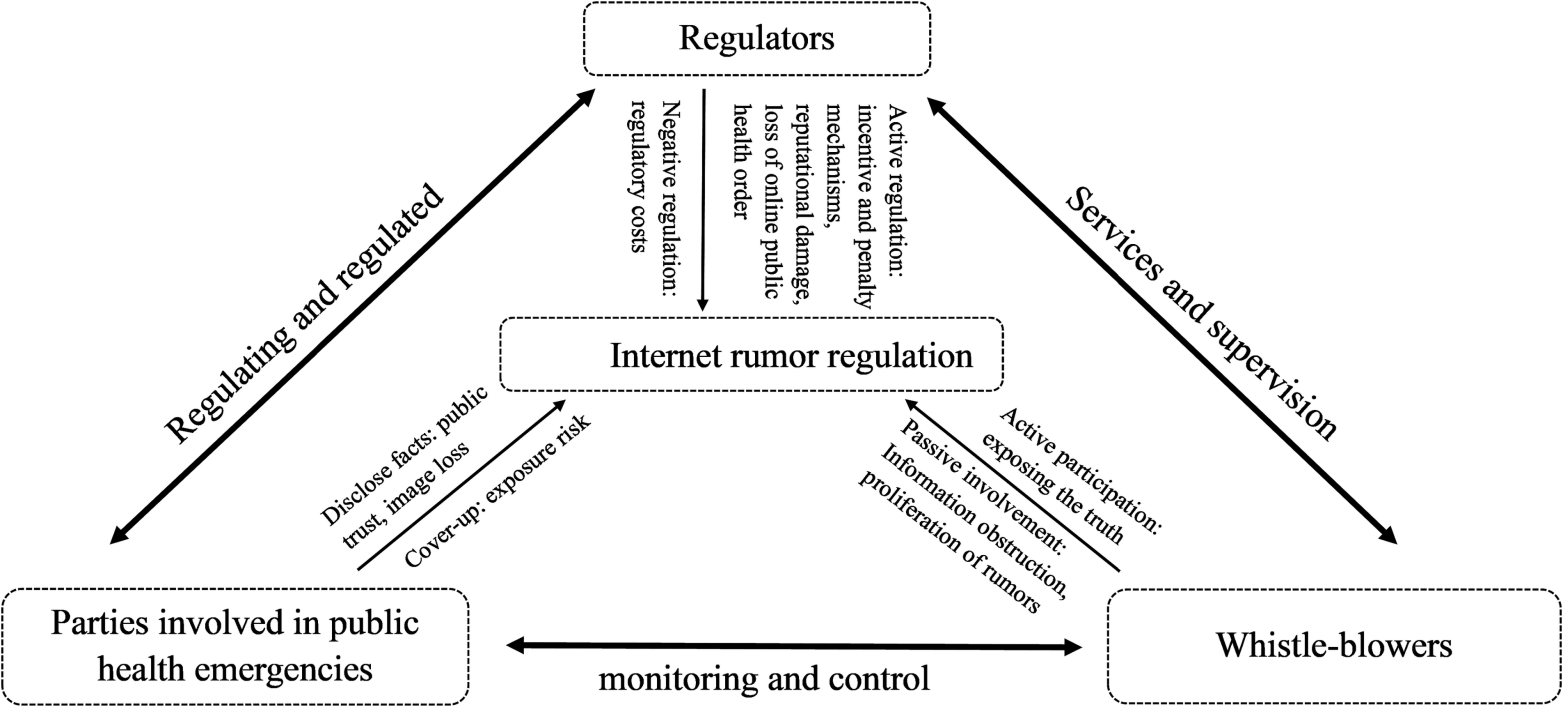
Evolutionary gaming system for rumor management of public health emergencies.

Therefore, to effectively regulate online rumors, it is necessary to establish a regulation model based on their triadic interactions. Such an approach has the potential to increase the efficiency of online rumor regulation, thereby promoting the robust development of digital public health within the online domain. Based on real-world problems and existing literature (28, 30, 31), we have attempted to construct this analytical framework. Figure 1 illustrates the triadic subject interaction of network rumor regulation, providing a visual representation of our approach.

### 2.2 Model assumptions

Based on the above analysis and the facts we have investigated, the following modeling assumptions can be made (Table 1).

**Table 1:** Parameters and their meanings.

Parameter	Meaning
*x*	Probability that the regulator chooses an active regulatory strategy
*y*	Probability that PIPHE chooses a strategy of disclosing facts
*z*	Probability that a whistle-blower chooses to actively participate in the regulator’s online rumor regulation
C	Regulatory costs paid by the regulator
*C_o_*	Fixed costs, the amount of money that the regulator needs to invest in the initial period of regulation
*H*	Variable costs, indicating the increase in costs as the regulatory mandate of the regulator increases
*T*	Reputation loss incurred by the regulator in the absence of regulation.
*R*	Fines imposed by the regulator on PIPHE
*D*	Image damage borne by PIPHE when they choose to disclose the facts strategy
*B*	Reputational damage when parties to a public health incident choose a cover-up strategy
*u*	Probability that PIPHE will be exposed for a cover-up
*m*	Probability that the regulator will expose the party involved in a public health emergencies
*n*	Probability that a whistle-blower will expose a party involved in a public health emergencies
*g*	Extent of the rumor spread
*k*	Hazard level of a public health emergencies
*U*	The elevation of social responsibility and civic awareness acquired by whistle-blowers upon choosing an active engagement
*E*	Costs of time and effort borne by whistle-blowers choosing to be actively involved
*A*	Regulatory rewards received by whistle-blowers for reporting rumors

*Assumption 1*: In the game model, the probability that the regulator opts for a proactive regulatory strategy is denoted as *x* (0 ≤ *x* ≤ 1), while the probability of selecting a passive regulatory strategy is 1 − *x*. The probability that the parties involved in public health incidents choose an open disclosure strategy is represented as *y* (0 ≤ *y* ≤ 1), whereas the probability of opting for a strategy of concealing facts is 1 − *y*. whistle-blowers exhibit a probability *z* (0 ≤ *z* ≤ 1) of actively engaging in regulatory oversight of online rumor regulation, and a probability of 1 − *z* for passive engagement. Similarly to the related research (25, 26), the variables *x*, *y*, and *z* vary over time, while the other variables remain constant.

*Assumption 2:* When the regulator opts for a proactive regulatory strategy, the regulatory cost *C* incurred consists of fixed costs and variable costs, where $C=C_{o}+H\;g$ . Here, *C_o_* and *H* are constants; *C_o_* represents the fixed costs, denoting the initial capital investment required by the regulators at the outset of supervision. This includes resource allocation for hardware, software, and personnel. *H* represents the variable costs, signifying the incremental costs that escalate in tandem with the augmentation of regulatory tasks undertaken by the regulators. Conversely, in the scenario where the regulator selects a passive regulatory strategy, its reputation loss is denoted as *T*. Additionally, the regulator is exposed to a loss in the network’s public health order, indicated as *g U*. When the PIPHE opt for a strategy of disseminating false information, and if exposed, the regulator imposes a fine denoted as *R*.

*Assumption 3*: The party involved in the public health incident needs to bear the image loss caused by the public health incident, denoted as *D*. When the party involved in the public health incident chooses the strategy of disclosing the facts, the reputation loss is represented as *B*. When the party involved in the public health emergencies chooses the strategy of covering up the facts and the regulator chooses the strategy of regulating the facts, the probability of the party involved in the public health incident’s covering up the facts being exposed with the participation of the netizens is denoted as u=m+n . This encompasses the probability of the regulator exposing parties in public health incidents (*m*) and the probability of whistle-blowers exposing parties (*n*).

*Assumption 4*: The extent of rumor propagation is denoted as *g*, and its magnitude is directly correlated with the level of active engagement by whistle-blowers, thereby influencing the strategic behaviors of the triadic entities. We postulate that the extent of rumor propagation is a function of the proportion of active engagement by whistle-blowers, i.e., $g=k\;\left(1-z\right)$ . When whistle-blowers opt for active engagement, they experience an augmentation of social responsibility and civic awareness denoted as *U*, while simultaneously incurring certain temporal and effort costs denoted as *E*. Additionally, engaging in whistle-blowing activities entitles whistle-blowers to a certain reward denoted as *A* from regulator.

A three-dimensional matrix to represent the payment matrix of the tripartite evolutionary game of collaborative governance among regulators, parties involved in public health emergencies and whistle-blowers in rumor dissemination of public health emergencies can be shown in the following Table 2.

**Table 2:** Payment matrix.

	Active regulation by regulators (*x*)		Negative regulation by regulators (1 − *x*)	
	Disclose facts by PIPHE (*y*)	Cover-up by PIPHE (1 − *y*)	Disclose facts by PIPHE (*y*)	Cover-up by PIPHE (1 − *y*)
Active participation of whistle-blowers (*z*)	$-C_{o}-H\;g\;k$	$-C_{o}-H\;gk+\left(m+n\right)\;R-n\;A$	−gkT	−gkT+nR
	−Dk	$-\left(m+n\right)\;R-gk\;B$	−Dk	−nR−gkB
	gkU−E	gkU−E+ngA	gkU−E	gkU−E
Negative participation by whistle-blowers (1 − *z*)	$-C_{o}-H\;g\;k$	$-C_{o}-H\;gk+m\;R$	−gkT	−gkT
	−Dk	−*m R* − *g k B*	−*D k*	−*g k B*
	0	0	0	0

## 3 Model analysis

### 3.1 Analysis of replication dynamics

Based on the above payment matrix, the equilibrium strategies of the regulator, PIPHE and whistle-blowers are further analyzed according to evolutionary game theory. Let the expected payoff of the regulator choosing the regulatory strategy be *U*_11_, the expected payoff of the regulator choosing the non-regulatory strategy be *U*_12_, and the average payoff be *U*_1_, then there is Eq. 1.

(1) $$\left\{ \begin{array}{l} U_{11}=y\left(\left(-C_{o}-gHk\right)\left(1-z\right)+\left(-C_{o}-gHk\right)z\right)+\\ \;\left(1-y\right)\left(\begin{array}{l} \left(-C_{o}-gHk+mR\right)\left(1-z\right)+\\ \left(-C_{o}-gHk-\mathrm{An}+\left(m+n\right)R\right)z \end{array}\right)\\ \begin{array}{l} U_{12}=y\left(-gkT\left(1-z\right)-\mathrm{gkTz}\right)+\left(1-y\right)\\ \left(-gkT\left(1-z\right)+\left(nR-gkT\right)z\right) \end{array}\\ U_{1}=xU_{11}+\left(1-x\right)U_{12} \end{array}\right.$$

Drawing from relevant research, the replicated dynamic equation for the regulator’s selection of a supportive strategy can be derived based on the principles of the Malthusian dynamic equation, as denoted by Eq. 2.

(2) $$F\left(x\right)=\frac{dx}{dt}=x\left(U_{11}-U_{1}\right)=\left(-1+x\right)x\left(\begin{array}{l} C_{o}+gk\left(H-T\right)\\ +\left(-1+y\right)\\ \left(mR-Anz\right) \end{array}\right)$$

Let the expected payoff of the PIPHE choosing the disclose facts strategy be *U*_21_, the expected payoff of the PIPHE choosing the cover-up strategy be *U*_22_, and the average payoff be *U*_2_, then there is Eq. 3.

(3) $$\left\{\begin{array}{l} U_{21}=\;\left(1-x\right)\left(-Dk\;\left(1-z\right)-Dkz\right)+x\left(-Dk\;\left(1-z\right)-Dkz\right)\\ U_{22}=x\left(\left(-Bgk-mR\right)\left(1-z\right)+\left(-Bgk+\left(-m-n\right)R\right)z\right)+\\ \;\left(1-x\right)\;\left(-Bgk\left(1-z\right)+\left(-Bgk-nR\right)z\right)\\ U_{2}=yU_{21}+\left(1-y\right)U_{22} \end{array}\right.$$

The replicated dynamic equation for the PIPHE choosing the disclose facts strategy is Eq. 4.

(4) $$F\left(y\right)=\frac{\mathrm{dy}}{dt}=y\left(U_{21}-U_{2}\right)=-\left(-1+y\right)y\left(\begin{array}{l} -Dk+Bgk\\ +mRx+nRz \end{array}\right)$$

Let the expected payoff of the whistle-blowers choosing the active participation strategy be *U*_31_, the expected payoff of the whistle-blowers choosing the negative participation strategy be *U*_32_, and the average payoff be *U*_3_, then there is Eq. 5.

(5) $$\left\{\begin{array}{l} U_{31}=\left(\left(-E-gkU\right)\left(1-x\right)+\left(-E+Agn+gkU\right)x\right)\;\left(1-y\right)+\\ \left(\left(-E+gkU\right)\left(1-x\right)+\left(-E+gkU\right)x\right)\;y\\ U_{32}=0\\ U_{3}=zU_{31}+\left(1-z\right)U_{32} \end{array}\right.$$

The replicated dynamic equation for the whistle-blowers choosing the active participation strategy is Eq. 6.

(6) $$\begin{array}{l} F\left(z\right)=\frac{dz}{dt}=z\left(U_{31}-U_{3}\right)=\left(-1+z\right)\\ \;z\left(E-gkU+\mathrm{Agnx}\left(-1+y\right)\right) \end{array}$$

### 3.2 Stable equilibrium analysis

The coupling of Eqs. 2, 4, 6 yields a three-dimensional dynamical system (I), i.e., Eq. 7.

(7) $$\left\{ \begin{array}{l} F\left(x\right)=\left(-1+x\right)x\left(C_{o}+gk\left(H-T\right)+\left(-1+y\right)\left(mR-Anz\right)\right)\\ F\left(y\right)=-\left(-1+y\right)y\left(-Dk+Bgk+mRx+nRz\right)\\ F\left(z\right)=\left(-1+z\right)z\left(E-gkU+\mathrm{Agnx}\left(-1+y\right)\right) \end{array}\right.$$

Let $\left(F\left(x\right),F\left(y\right),F\left(z\right)\right)=\left(\frac{dx}{dt},\frac{\mathrm{dy}}{dt},\frac{dz}{dt}\right)=\left(0,0,0\right)$ , we can get *E*_1_ (0,0,0), *E*_2_ (1,0,0), *E*_3_ (0,1,0), *E*_4_ (0,0,1), *E*_5_ (1,1,0), *E*_6_ (1,0,1), *E*_7_ (0,1,1), *E*_8_ (1,1,1), and *E*_9_ (x*,y*,z*). *E*_9_ is meaningful under certain conditions; it is not a pure strategy equilibrium. If the equilibrium of the three-party evolutionary game is an asymptotically stable state, the equilibrium must be a strict Nash equilibrium, which is a pure strategy equilibrium. Therefore, the asymptotic stability of the three-party evolutionary game only needs to discuss the asymptotic stability of the pure strategy equilibrium point in the replication dynamic equation, that is, discuss the asymptotic stability of *E*_1_~*E*_8_ (34–36). The aforementioned equilibrium points may not necessarily constitute evolutionary stable strategies (ESS) within the evolutionary game system. Therefore, it is essential to further examine whether these stable points indeed represent stable strategies and to identify the conditions under which they qualify as stable strategies.

First, the asymptotic stability of the above eight equilibrium is further discriminated by the local stability of the Jacobi matrix. The Jacobi matrix of the game equations is obtained by taking the first-order partial derivatives of *F*(*x*), *F*(*y*), and *F*(*z*) concerning *x*, *y*, and *z*, then there is Eq. 8.

(8) $$J=\left[\begin{array}{ccc} F_{x}\left(x\right) & F_{y}\left(x\right) & F_{z}\left(x\right)\\ F_{y}\left(y\right) & F_{y}\left(y\right) & F_{y}\left(y\right)\\ F_{z}\left(z\right) & F_{z}\left(z\right) & F_{z}\left(z\right) \end{array}\right]$$

According to the Lyapunov theory, when all eigenvalues of the Jacobian matrix, denoted as *λ*, are less than zero, the point is asymptotically stable. Conversely, when all eigenvalues of the Jacobian matrix are greater than zero, the point is unstable. When the eigenvalues of the Jacobian matrix exhibit a mix of positive and negative values, the equilibrium point is unstable, referred to as a saddle point. The asymptotic stability analysis of equilibrium points is presented in Table 3. Under specific conditions, each of the eight stable points possesses asymptotic evolutionary stability. These conditions are as follows:

**Table 3:** Asymptotic stability analysis of local equilibrium points.

Equilibrium	Eigenvalue (math.)	In the end
(0,0,0)	$\lambda_{1}=-C_{o}+m\;R-gk\;\left(H-T\right)$	Stable point when $-C_{o}+m\;R-gk\;\left(H-T\right)<0$ , −Dk+Bgk<0 , and −E+gkU<0 , otherwise saddle point or unstable point
	$\lambda_{2}=-D\;k+B\;g\;k$	
	$\lambda_{3}=-E+gkU$	
(1,0,0)	$\lambda_{1}=C_{o}-m\;R+gk\;\left(H-T\right)$	Stable point when $C_{o}-m\;R+gk\;\left(H-T\right)<0$ , −Dk+Bgk+mR<0 , and −E+Agn+gkU<0 , otherwise saddle or unstable point
	$\lambda_{2}=-D\;k+B\;gk+m\;R$	
	$\lambda_{3}=-E+A\;g\;n+gkU$	
(0,1,0)	$\lambda_{1}=-C_{o}-gk\;\left(H-T\right)$	Stable point when $-C_{o}-gk\;\left(H-T\right)<0$ , Dk−Bgk<0 , and −E+gkU<0 , otherwise saddle or unstable point
	$\lambda_{2}=D\;k-B\;g\;k$	
	$\lambda_{3}=-E+gkU$	
(0,0,1)	$\lambda_{1}=-C_{o}-A\;n+m\;R-gk\;\left(H-T\right)$	Stable point when $-C_{o}-A\;n+m\;R-gk\;\left(H-T\right)<0$ , −Dk+Bgk+nR<0 , and E−gkU<0 otherwise saddle point or unstable point
	$\lambda_{2}=-D\;k+B\;gk+n\;R$	
	$\lambda_{3}=E-gkU$	
(1,1,0)	$\lambda_{1}=C_{o}+gk\;\left(H-T\right)$	Stable point when $C_{o}+gk\;\left(H-T\right)<0$ , Dk−Bgk−mR<0 , and −E+gkU<0 otherwise saddle point or unstable point
	$\lambda_{2}=D\;k-B\;g\;k-m\;R$	
	$\lambda_{3}=-E+gkU$	
(1,0,1)	$\lambda_{1}=C_{o}+A\;n-m\;R+gk\;\left(H-T\right)$	Stable point when $C_{o}+A\;n-m\;R+gk\;\left(H-T\right)<0$ , $-D\;k+B\;gk+\left(m+n\right)\;R<0$ , and E−Agn−gkU<0 , otherwise saddle point or unstable point
	$\lambda_{2}=-D\;k+B\;gk+\left(m+n\right)\;R$	
	$\lambda_{3}=E-A\;g\;n-gkU$	
(0,1,1)	$\lambda_{1}=-C_{o}-gk\;\left(H-T\right)$	Stable point when $-C_{o}-gk\;\left(H-T\right)<0$ , Dk−Bgk−nR<0 , and E−gkU<0 otherwise saddle point or unstable point
	$\lambda_{2}=D\;k-B\;g\;k-n\;R$	
	$\lambda_{3}=E-gkU$	
(1,1,1)	$\lambda_{1}=C_{o}+gk\;\left(H-T\right)$	Stable point when $C_{o}+gk\;\left(H-T\right)<0$ , $D\;k-B\;g\;k-\left(m+n\right)\;R<0$ , and E−gkU<0 , otherwise saddle or unstable point
	$\lambda_{2}=D\;k-B\;g\;k-\left(m+n\right)\;R$	
	$\lambda_{3}=E-gkU$	

*Scenario 1*: *E*_1_ (0,0,0) is the stable equilibrium when $-C_{o}+m\;R-g\;k\;\left(H-T\right)<0$ , −Dk+Bgk<0 , and −E+gkU<0 . At this point, the regulatory cost of the regulator is higher than its reputation loss and penalty income, and the image loss of the PIPHE is higher than its reputation enhancement and penalty expenditure, and the cost of the whistle-blower’s time and effort is higher than his or her social responsibility and reward income. In such a scenario, this equilibrium is detrimental to the prompt detection and management of public health incidents, as well as to the safeguarding of public awareness and a sense of security. Rumors may spread widely on the Internet, resulting in social instability and panic. Therefore, there is a need to break this equilibrium through measures such as improving the accountability and capacity of the regulator, increasing the transparency and integrity of the PIPHE, and encouraging increased participation and protection for whistle-blowers.

*Scenario 2*: *E*_2_ (1,0,0) is a stable equilibrium when $C_{o}-m\;R+g\;k\;\left(H-T\right)<0$ , −Dk+Bgk+mR<0 , and −E+Agn+gkU<0 . At this point, the cost of regulation to the regulator is lower than its reputation loss and penalty income, the image loss of the PIPHE is lower than its reputation enhancement and penalty expenditure, and the cost of the whistle-blower’s time and effort is higher than its social responsibility and reward income. In this scenario, this equilibrium favors the functioning of the regulator, yet it also presents certain issues. On the one hand, if the regulatory capacity of the regulator is inadequate or subject to interference, the parties involved in a public health incident may evade punishment for concealing facts, leading to the spread of rumors and social distrust. On the other hand, insufficient engagement of whistle-blowers could result in regulatory authorities lacking effective information sources and social support, thereby diminishing regulatory efficacy. Therefore, there is a need to improve this balance through measures such as increasing the legal responsibility of the PIPHE and the penalties for moral hazard and increasing the incentivization mechanisms and safeguards for whistle-blowers.

*Scenario 3*: *E*_3_ (0,1,0) is a stable equilibrium when $-C_{o}-g\;k\;\left(H-T\right)<0$ , Dk−Bgk<0 , and −E+gkU<0 . At this point, the regulatory cost of the regulator is higher than its reputation loss and penalty income, the image loss of the person involved in the public health incident is lower than its reputation enhancement and penalty expenditure, and the cost of the whistle-blower’s time and effort is higher than its social responsibility and reward income. The equilibrium in this scenario is favorable to the PIPHE demonstrating its integrity and transparency, but there are some risks. On the one hand, if the public health emergency party’s disclosure of facts is incomplete or untrue, then negative regulation by the regulator may lead to the generation and spread of rumors, affecting the trust and safety of the community. On the other hand, inadequate engagement of whistle-blowers might result in a lack of effective validation and feedback for the disclosed facts by the PIPHE, thereby causing distortion and misinformation of information. Hence, it is necessary to optimize this equilibrium through certain measures, such as enhancing the sense of responsibility and capacity of regulators, and augmenting incentivization mechanisms and safeguards for whistle-blowers.

*Scenario 4*: *E*_4_ (0,0,1) is a stable equilibrium when $-C_{o}-A\;n+m\;R-g\;k\;\left(H-T\right)<0$ , −Dk+Bgk+nR<0 , and E−gkU<0 . At this point, the regulatory cost of the regulator is higher than its reputation loss and penalty income; the image loss of the PIPHE is higher than its reputation enhancement and penalty expenditure; the cost of the whistle-blower’s time and effort is lower than its social responsibility and reward income. In this scenario, the equilibrium is conducive to the role of whistle-blowers; however, it also presents certain challenges. On one hand, if regulators’ passive oversight leads to untimely and ineffective handling of whistle-blowers’ reports, the participation of whistle-blowers could be hindered and discouraged, potentially fostering the spread of rumors and societal distrust. On the other hand, if the concealment of facts by the PIPHE results in insufficient and authentic evidence for whistle-blowers’ reports, the involvement of whistle-blowers may face scrutiny and backlash, causing distortion and misguidance of information. Therefore, measures need to be implemented to enhance this equilibrium, such as reinforcing the sense of responsibility and capacity of regulatory authorities, enhancing transparency and integrity of the PIPHE, and safeguarding the rights and security of whistle-blowers.

*Scenario 5*: *E*_5_ (1,1,0) is a stable equilibrium when $C_{o}+g\;k\;\left(H-T\right)<0$ , Dk−Bgk−mR<0 , and −E+gkU<0 . At this point, the cost of regulation to the regulator is lower than its reputation loss and penalty income, the image loss of the PIPHE is higher than its reputation enhancement and penalty expenditure, and the cost of the whistle-blower’s time and effort is higher than its social responsibility and reward income. The equilibrium in this scenario is favorable for regulators and PIPHE to demonstrate their integrity and transparency, yet it also presents certain limitations. On the one hand, if the regulator’s supervisory capacity is insufficient or interfered with, then the public facts of PIPHE may lack effective verification and feedback, leading to distortion and misdirection of information. On the other hand, if the participation level of whistle-blowers is too low, then the public facts of the regulatory authorities and the parties involved in the public health incident may lack effective sources of information and social support, leading to the generation and dissemination of rumors. Therefore, there is a need to optimize this equilibrium through various measures, such as enhancing the sense of responsibility and capacity of the regulator, and implementing incentive mechanisms and safeguards for whistle-blowers, among others.

*Scenario 6*: *E*_6_ (1,0,1) is a stable equilibrium when $C_{o}+A\;n-m\;R+g\;k\;\left(H-T\right)<0$ , $-D\;k+B\;g\;k+\left(m+n\right)\;R<0$ , and E−Agn−gkU<0 . At this point, the cost of regulation to the regulator is lower than its reputation loss and penalty income, the image loss of the PIPHE is lower than its reputation enhancement and penalty expenditure, and the cost of the whistle blower’s time and effort is lower than its social responsibility and reward income. The equilibrium in this scenario is favorable for regulators and whistle-blowers to perform their roles, but there are some challenges. On the one hand, if the regulator’s regulatory capacity is insufficient or interfered with, whistle-blowers’ reports may not be dealt with in a timely and effective manner, leading to the spread of rumors and social distrust. On the other hand, if the cover-up by the parties involved in public health emergencies results in whistle-blowers’ reports not being supported by sufficient and truthful evidence, then whistle-blowers’ participation may be challenged and attacked, leading to distorted and misleading information. Therefore, it is imperative to enhance this equilibrium through various measures, such as enhancing the accountability and capacity of the regulatory authorities, increasing transparency and integrity of the PIPHE, and protecting the rights and security of whistle-blowers.

*Scenario 7*: *E*_7_ (0,1,1) is a stable equilibrium when $-C_{o}-g\;k\;\left(H-T\right)<0$ , Dk−Bgk−nR<0 , and E−gkU<0 . At this point, the regulatory cost of the regulator is higher than its reputation loss and penalty income, the image loss of the PIPHE is lower than its reputation enhancement and penalty expenditure, and the cost of the whistle-blower’s time and effort is lower than its social responsibility and reward income. The equilibrium in this scenario is conducive to the PIPHE and whistle-blowers demonstrating their integrity and transparency, but there are some limitations. On one hand, if passive regulatory oversight by the regulator results in a lack of effective validation and feedback for the public disclosures made by the PIPHE, distortions and misinterpretations of information may lead to the generation and dissemination of rumors. On the other hand, if the engagement level of whistle-blowers remains insufficient, the publicly disclosed facts by both the PIPHE and regulator might lack robust sources of information and societal support, potentially giving rise to uncontrolled public opinion and conflicts. Therefore, it becomes necessary to optimize this equilibrium through various measures, such as enhancing the sense of responsibility and capacity of the regulator, introducing incentives and protective mechanisms for whistle-blowers, and other strategies.

*Scenario 8*: *E*_8_ (1,1,1) is a stable equilibrium when $C_{o}+g\;k\;\left(H-T\right)<0$ , $D\;k-B\;g\;k-\left(m+n\right)\;R<0$ , and E−gkU<0 . At this point, the cost of regulation to the regulator is lower than its reputation loss and penalty income, the image loss of the PIPHE is higher than its reputation enhancement and penalty expenditure, and the cost of the whistle blower’s time and effort is lower than its social responsibility and reward income. The equilibrium in this scenario is favorable for regulators, the PIPHE and whistle-blowers to perform their roles, but there are some challenges. On the one hand, if the regulator’s regulatory capacity is insufficient or interfered with, the public facts of the PIPHE and the whistle-blower’s report may not be dealt with in a timely and effective manner, leading to the spread of rumors and social distrust. On the other hand, if the public facts of the parties involved in public health emergencies and the whistle-blower’s report cannot be fully and truthfully substantiated, then the behavior of the regulator and the whistle-blower may be questioned and attacked, leading to distorted and misleading information. Therefore, there is a need to improve this equilibrium through several measures, such as improving the accountability and capacity of the regulator, increasing the transparency and integrity of the PIPHE, and protecting the rights and security of whistle-blowers.

## 4 Numerical modeling

To further verify the correctness of the model derivation and the reasonableness of the discussion, dynamic evolutionary simulations of the gaming system were conducted using Matlab. Based on the above evolutionary game analysis, it can be found that active regulation by the regulator, active disclosure of facts by the PIPHE, and active participation by the whistle-blowers are the most realistic ideal ESS. Based on the assumptions, numerical simulations were performed using the ideal ESS as a benchmark scenario. In this context, it is necessary to satisfy the conditions $C_{o}+g\;k\;\left(H-T\right)<0$ , $D\;k-B\;g\;k-\left(m+n\right)\;R<0$ , and E−gkU<0 , so the parameters can be set as follows: *Co =* 4; *H =* ***3***; *T =* 5; *R =* 4; *D =* 4; *B =* 4; *u =* 0.6; *m =* 0.4; *n =* 0.4; *g =* 0.8; *k =* 3; *U =* 2; *E =* 4; *A =* 3; *x* = 0.6, *y* = 0.5, and *z* = 0.7, and the simulation period *t is* set to 10.

### 4.1 Impact of regulator’s behavior

**Figure 2:**
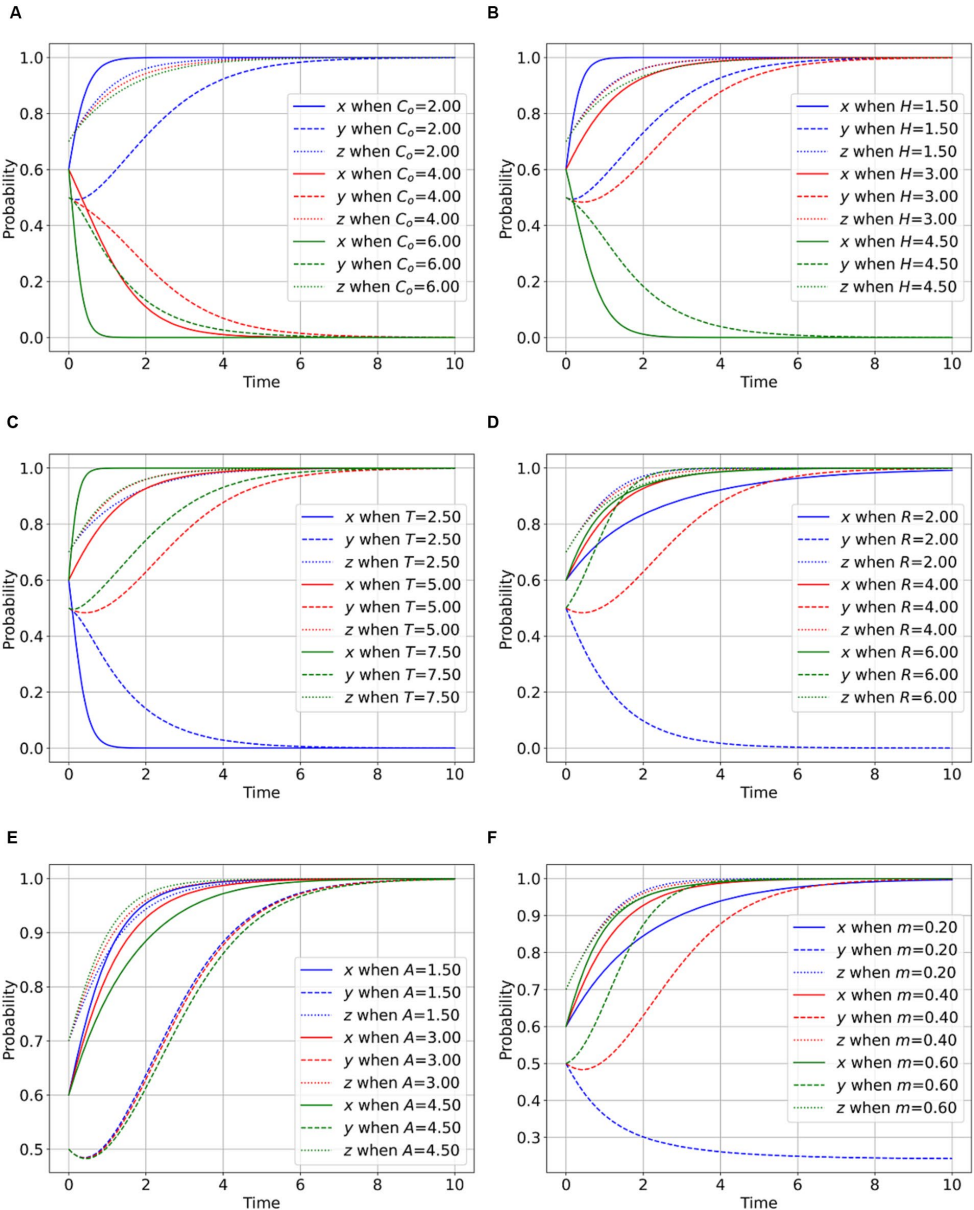
Impact of the regulator’s behavioral parameters on the evolutionary game. **(A)** Impact of *C_o_* on the player behavior. **(B)** Impact of *H* on the player behavior. **(C)** Impact of *T* on the player behavior. **(D)** Impact of *R* on the player behavior. **(E)** Impact of *A* on the player behavior. **(F)** Impact of *m* on the player behavior.

As shown in Figures 2A–C, the higher the regulatory cost paid by the regulator, the lower the willingness of the regulator to actively regulate, and the cost of regulation inhibits the regulator’s motivation. When the cost becomes excessively high, the regulator may become disinclined to engage in regulation. The willingness of the parties involved in public health emergencies to disclose the facts decreases, and the enthusiasm of whistle-blowers for active supervision diminished. As illustrated in Figure 2D, an increase in the penalty imposed on PIPHE by the regulator enhances the willingness of PIPHE to disclose factual information. This finding suggests that higher penalties serve to amplify the motivation of parties involved in public health emergencies to disclose information, thereby stimulating proactive regulatory actions. In Figure 2E, a greater reward offered by regulators to whistle-blowers for reporting leads to an increased inclination of whistle-blowers to participate actively in rumor management. This observation underscores the incentivizing effect of rewards in motivating whistle-blowers to engage more actively in rumor control efforts. As in Figure 2F, a higher probability of the regulator exposing parties involved in public health emergencies corresponds to an elevated willingness of PIPHE to disclose facts. This outcome signifies that the proactive nature and capabilities of the regulator can encourage parties involved in public health emergencies to choose behavior that involves disclosing facts.

### 4.2 The impact of the behavior of those involved in a public health incident

**Figure 3:**
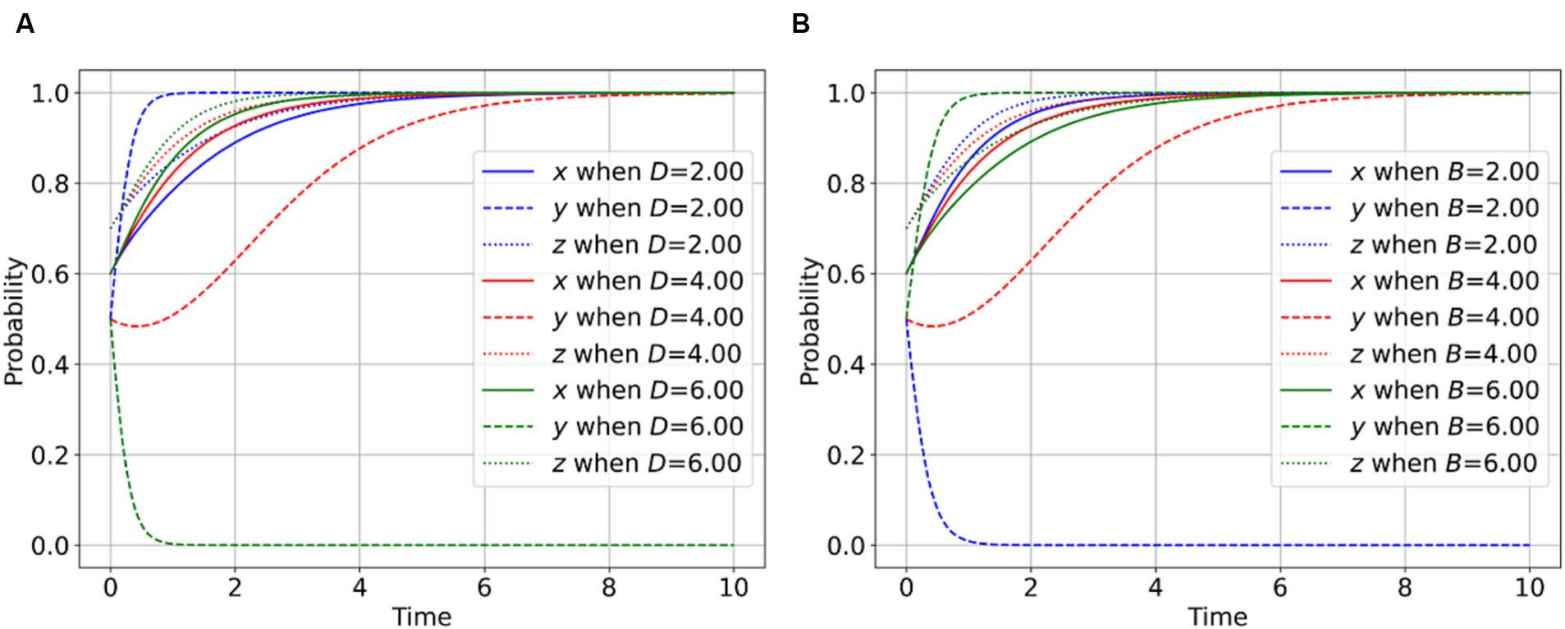
Effect of behavioral parameters of PIPHE on the evolutionary game. **(A)** Impact of *D* on the player behavior. **(B)** Impact of *B* on the player behavior.

As illustrated in Figure 3A, a higher degree of image loss incurred by parties involved in public health emergencies due to disclosing facts is associated with a reduced willingness to disclose facts. This observation suggests that the potential for image loss acts as a deterrent to the integrity-driven behavior of parties involved in public health emergencies. In this scenario, there is an increased inclination of regulators to engage in regulation, and correspondingly, a heightened willingness of whistle-blowers to participate in supervision efforts. In Figure 3B, an increase in the reputation gain acquired by PIPHE through the disclosure of facts corresponds to an augmented motivation for PIPHE to exhibit greater transparency and candor. This outcome implies that the prospect of reputation enhancement serves as an incentive for parties involved in public health emergencies to be more forthcoming and transparent. Given the intrinsic good self-discipline of stakeholders in public health incidents, wherein instances of concealing facts are infrequent or minimal, the safeguarding of the public’s right to be informed is effectively ensured. Consequently, in such instances, the willingness of regulators to engage in regulation tends to decrease, while the willingness of whistle-blowers to participate in supervision efforts tends to increase.

### 4.3 Impact of whistle-blower’s behavior

**Figure 4:**
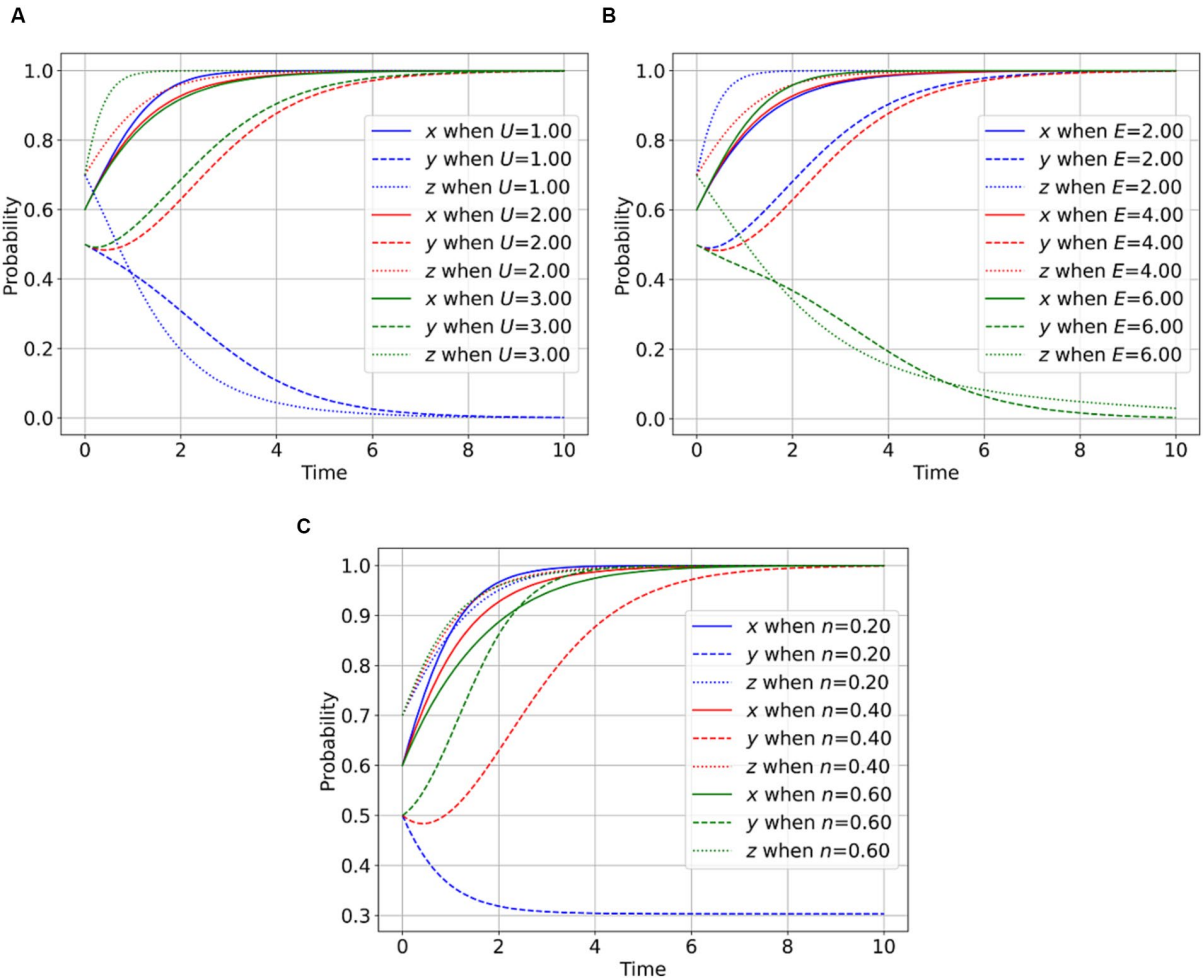
Effect of whistle blower’s behavioral parameters on the evolutionary game. **(A)** Impact of *U* on the player behavior. **(B)** Impact of *E* on the player behavior. **(C)** Impact of n on the player behavior.

As depicted in Figure 4A, a higher magnitude of psychological gain derived by whistle-blowers from an increased sense of social responsibility and civic awareness, resulting from their act of reporting, corresponds to an elevated willingness of whistle-blowers to engage in supervision. This observation underscores that the enhancement of social responsibility and civic awareness serves as a motivating factor for whistle-blowers to proactively participate in the management of rumors. In this scenario, the inclination of the regulator toward proactive regulation is reduced, while the willingness of parties involved in public health emergencies to disclose facts is heightened. In Figure 4B, an increase in the time and effort costs borne by whistle-blowers because of their reporting activities corresponds to a diminished inclination of whistle-blowers to engage in supervision. This finding indicates that the time and effort costs exert a dampening effect on the enthusiasm of whistle-blowers. Consequently, the willingness of regulators to engage in proactive regulation increases, while the willingness of parties involved in public health emergencies to disclose facts diminishes. In Figure 4C, a higher probability of whistle-blowers exposing parties involved in public health emergencies corresponds to an augmented willingness of parties involved in public health emergencies to disclose facts. This outcome highlights that the initiative and capability of whistle-blowers can ensure effective social oversight, thereby driving regulators and parties involved in public health emergencies toward greater integrity and transparency through the revelation of truths.

### 4.4 Impact of other exogenous factors

**Figure 5:**
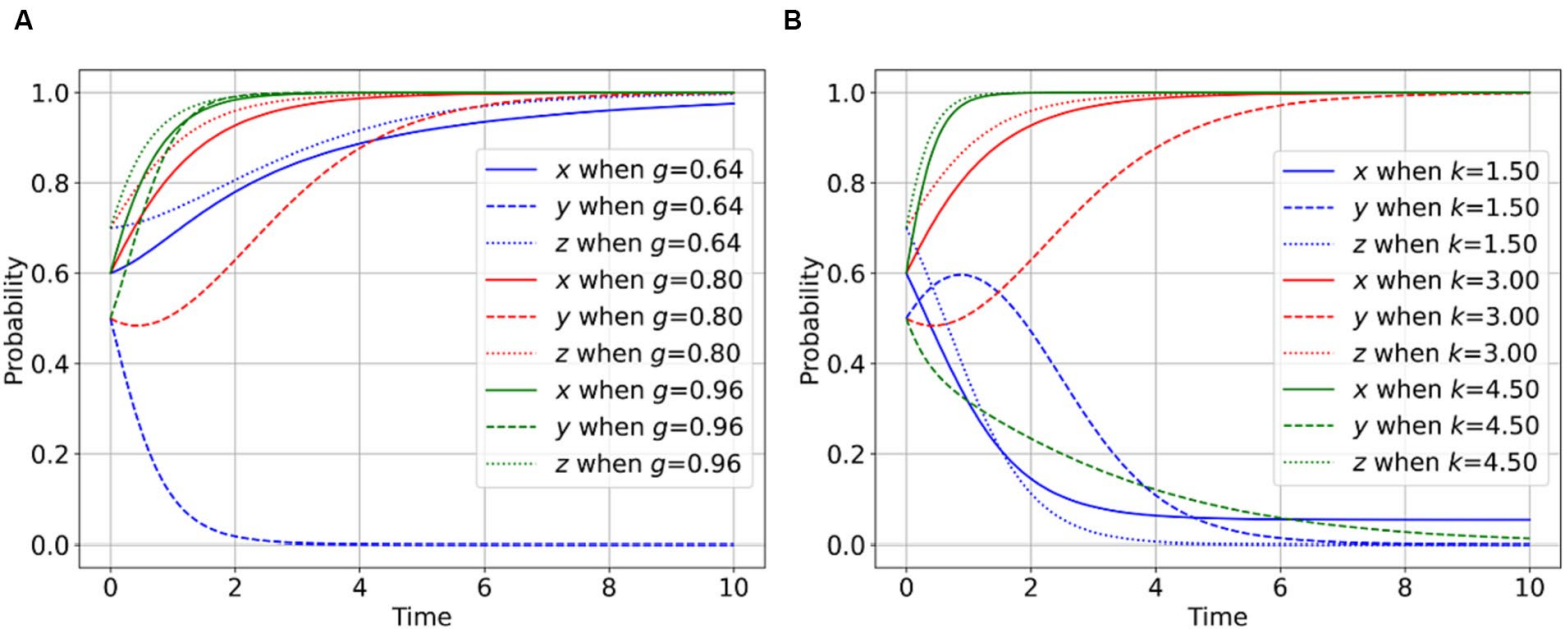
Impact of other exogenous factors on the evolutionary game. **(A)** Impact of *g* on the player behavior. **(B)** Impact of *k* on the player behavior.

As shown in Figure 5A, a higher magnitude of rumor propagation extent corresponds to an increased number of concealed facts perpetuated by the rumor, resulting in escalated reputational risks. At this time, the regulator, PIPHE and the whistle-blowers all have a stronger willingness to participate in the rumor management of the public health emergencies, i.e., the regulator’s willingness to actively regulate is stronger, and the willingness of PIPHE to disclose the facts is stronger, and the whistle-blower’s willingness to participate in the supervision is more advanced. As shown in Figure 5B, a higher level of severity denoted by parameter in public health incidents corresponds to greater societal losses caused by the propagation of rumors. In such circumstances, regulators, PIPHE, and whistle-blowers all exhibit a more pronounced willingness to engage in the management of rumors associated with public health incidents.

## 5 Discussion

The results of the model analysis in this paper show that rumor spreading and its collaborative governance in public health emergencies is a dynamic evolutionary process, which is affected by a variety of factors, such as regulatory costs, fines, incentives, image loss, credibility enhancement, and exposure probability. These factors affect the behavioral strategies and game results of all parties. The findings of the model analysis in this study exhibit a degree of congruence with empirical cases, for example:

In the Fukushima nuclear leak incident in 2011, as the Japanese government and TEPCO did not disclose the truth and impact of the accident promptly, various rumors appeared on the Internet, such as “iodized salt can protect against radiation,” “Fukushima nuclear leak will contaminate seawater and lead to an increase in the price of sea salt,” etc., triggering a “salt rush.” and so on, which triggered the “salt rush.” This is consistent with the results of the model analysis conducted in this paper, wherein it is demonstrated that the adoption of the strategy by PIPHE to conceal facts contributes to the proliferation of rumors and the instigation of societal panic.

In the 2015 Tianjin Port explosion incident, due to the delayed dissemination of official information, the public’s eager anticipation for the latest updates prompted them to seek alternative sources of information, thereby creating opportunities for the propagation of online rumors. During that time, rumors circulated on the internet suggesting that harmful substances might be blown toward Beijing, that there were numerous casualties at the scene, and a significant leakage of sodium chloride could result in widespread casualties. Additionally, some netizens speculated that the explosion in Tanggu was related to terrorists and that the responsible individual was the son of a deputy mayor. These instances align with the findings of the present study’s model analysis, which demonstrates that the adoption of a passive regulatory strategy by regulators can lead to the proliferation of rumors and societal instability.

In the 2020 COVID-19 pandemic, the outbreak and spread of the virus led to various rumors circulating on the internet regarding its origin, transmission pathways, prevention, and treatment methods. Examples of such rumors included claims that the novel coronavirus was artificially created, that it could be transmitted through mosquitoes, and that consuming alcohol could kill the virus. These rumors caused public panic and misinformation. In this process, there are some healthcare workers and scientists who act as whistle-blowers and expose the truth of the epidemic to the regulators or the media promptly, such as Dr. Li Wenliang and Academician Zhong Nanshan, etc. Their whistleblowing behaviors promote the active regulation of the regulator and the disclosure of the facts by the PIPHE, which effectively curbed the dissemination of the rumors. These findings align with the outcomes of the current study’s model analysis, affirming that whistle-blowers opting for active supervisory strategies can enhance the synergistic effectiveness of rumor control.

These cases show that in the governance of public opinion on ecological public health public opinion events, the tripartite subjects of the regulators, PIPHE, and the whistle-blowers play distinct roles and functions, each facing unique challenges and dilemmas. Coordinating the relationship between the three main parties, striking a balance between information disclosure and the demands of social stability, is conducive to advancing the management of rumors in public health incidents, and holds significant implications for addressing rumors of other types. This also, on the other hand, validates the significance of this study.

Compared to general rumor propagation, rumors in public health incidents exhibit certain distinct characteristics. On the one hand, public health emergencies involve people’s life safety, physical health, ecological public health and other important areas, once false information or rumors appear, it may trigger social panic, medical squeeze and other undesirable consequences to the people’s health and risk management with serious consequences (3), and even lead to social unrest and public order chaos. Therefore, public health emergency rumor governance requires a more timely, accurate and authoritative release of the truth mitigating information voids and misguidance. On the other hand, public health emergency rumors are usually related to people’s health and safety (37), making public health emergency rumors more likely to trigger emotional responses from the public (38), and more difficult to be identified and verified. Simultaneously, public health emergency rumors are also influenced and manipulated by multi-interested subjects, such as regulators, enterprises, media, and the public. These entities may report, comment on, or propagate public health emergencies for varying motivations and objectives, leading to the distortion or misrepresentation of information. For example, a series of coupling problems such as uncontrolled rumors and public psychological imbalance will always occur on social media, which brings great interference to crisis disposal (39). And the endogenous demand for health information generated by the public due to the lack of scientific knowledge of health information stimulates the dissemination of health information by mass media, and at the same time provides rumor mongers with the opportunity to publish and disseminate online rumors (19).

Difficulties in the governance of rumors about public health emergencies mainly involve information asymmetry, multiple interests and social trust. Firstly, information asymmetry is the main problem in the governance of public health emergencies because the complexity and multidimensionality of public health issues make it difficult for the public to obtain accurate and comprehensive information (40), and there may be biases and misunderstandings during the process of information acquisition, translation, and dissemination, which may promote the dissemination and spread of rumors. Second, multiple interests are the main reason for the differences and conflicts among stakeholders in public health emergency governance (41). Stakeholders such as regulators, enterprises, the public, and the media usually have different goals, priorities, and positions, and these factors may affect their perceptions, attitudes, and actions toward public health emergencies, thus increasing the difficulty of investigating, verifying, and handling the news. Third, the significant challenge to societal trust has emerged as a crucial impediment in the governance of public event rumors. With the application of generative AI tools, unverified, false or misleading information has reached unprecedented levels (42), and audiences have difficulty in identifying true and false viral content, resulting in social trust challenges that will continue to increase (43). Consequently, addressing the propagation of rumors in public health emergencies necessitates collaborative efforts among stakeholders such as regulators, parties, and the public. The convergence of social consensus and collaborative actions is essential to drive effective governance of public health concerns (41).

This study elucidates the collaborative behaviors of regulators, PIPHE, and whistle-blowers in the governance of rumors in public health emergencies. Several valuable insights have been derived: rumor spreading is a complex problem, which requires the participation of multiple parties, including regulators, PIPHE, whistle-blowers, and the media. Regulators should actively supervise, disclose information promptly (44), stop the spread of rumors and guide the public to take rational actions (45). The parties involved in public health emergencies should disclose the facts and fully disclose relevant information to win public recognition and trust. Whistle blowers should actively participate in supervision, expose the truth in time and avoid the spread of rumors. The media should guide public opinion, publish true information, dispel rumors to clarify misinformation and expose unfavorable rumors. Therefore, regulators should flexibly adjust their regulatory strategies according to different situations and stages, and reasonably set up incentives such as costs, fines and rewards to promote cooperation and coordination among all parties. At the same time, regulators should also pay attention to collecting and analyzing behavioral data and feedback from all parties to promptly identify problems and enhance methodologies.

Based on the above analysis, we can derive the following recommendations for rumor management in public health emergencies. Firstly, it is recommended to establish effective collaborative mechanisms among regulators, stakeholders in public health emergencies, and informants, such as information sharing, building trust, and incentivizing protection, in order to enhance the efficiency and effectiveness of rumor management. Secondly, it is advised to enhance the legal regulations and social norms pertaining to public health emergencies, including the formulation and enforcement of laws and regulations related to rumors, strengthening supervision and punishment of parties involved in public health emergencies, and protecting the legitimate rights and safety of informants, to enhance the legitimacy and credibility of rumor management. Lastly, it is suggested to utilize social media and digital technology to enhance the capability and level of rumor management, such as using big data and artificial intelligence for rumor detection and refutation, leveraging social media and online communities for rumor dissemination and supervision, and utilizing mobile applications and cloud services for rumor management and collaboration, in order to improve the timeliness and inclusiveness of rumor management.

## 6 Conclusion

This paper focuses on the dissemination of rumors related to public health emergencies on social media and their collaborative governance. By employing evolutionary game theory, a dynamic game model involving regulators, parties involved in public health emergencies, and whistle-blowers is constructed. This model is utilized to analyze the behavioral strategies and game outcomes of each party during the rumor propagation process. The effectiveness and feasibility of the model are verified through numerical simulations. Subsequently, the study explores the influencing factors of rumor propagation and proposes optimization strategies for collaborative governance involving regulators and the media. Based on the model analysis and numerical simulation, this paper draws the following conclusions:

1. There are a variety of possible equilibrium strategies between regulators, PIPHE and whistle-blowers. Among these, the most practically significant is the ideal Evolutionarily Stable Strategy (ESS) characterized by proactive regulation by regulatory authorities, truthful disclosure by public health stakeholders, and active engagement by whistle-blowers. To achieve this equilibrium, certain conditions need to be met, i.e., the parameters of regulatory costs, image loss, credibility enhancement, penalties, and incentives need to be within a reasonable range.
2. The higher the regulatory costs paid by the regulator, the lower the willingness of the regulator to actively regulate. Similarly, an increase in the fines imposed on PIPHE by regulators corresponds to a higher willingness on the part of PIPHE to disclose accurate information. Furthermore, greater rewards provided by regulators to whistle-blowers for reporting lead to a heightened willingness on the part of whistle-blowers to actively participate in oversight. Moreover, an elevated probability of the regulator exposing PIPHE’s actions results in a greater inclination of PIPHE to disclose truthful information.
3. The higher the image loss borne by the party involved in the public health incident due to disclosure of the facts, the lower the willingness of the party involved in the public health incident to disclose the facts; conversely, the higher the credibility enhancement gained by the party involved in the public health emergencies due to disclosure of the facts, the higher the willingness of the party involved in the public health incident to disclose the facts. Additionally, as the time and effort costs increase for whistle-blowers who actively engage, their willingness to participate in oversight decreases.
4. The degree of rumor spreading is directly correlated to the degree of active participation by whistle-blowers; as the extent of rumor propagation increases, the willingness of whistle-blowers to engage in oversight decreases, and vice versa. The degree of rumor propagation is influenced by factors such as the severity of public health risks associated with the event and the number of whistle-blowers involved.

This study addresses the deficiencies in existing literature and further enriches the network rumor regulation system, offering significant theoretical value. However, this study also has certain limitations. On the one hand, the model assumes that the three parties’ behavioral strategies are binary, that is, either positive or negative. In reality, a broader range of strategy options and combinations may exist. For instance, regulatory agencies may adopt different monitoring measures and intensities, stakeholders in public health events may utilize various information disclosure and crisis management techniques, and whistleblowers may use different reporting channels and methods. Therefore, future research could consider introducing more strategy variables and parameters to improve the model’s fit with real-world situations. On the other hand, the model only considers the impact of rumor propagation on the three parties’ behavioral choices and does not take into account the impact on the public and other stakeholders, such as media and digital platforms. Hence, future research could incorporate other relevant actors to provide a more comprehensive model.

## Data availability statement

The original contributions presented in the study are included in the article/supplementary material, further inquiries can be directed to the corresponding authors.

## Author contributions

YW designed the research framework and methodology. YW and LQ constructed the game model and simulation analyses. YW and SC was responsible for the literature analysis and strategy research. YW and LQ edited the manuscript. All authors contributed to the article and approved the submitted version.
