# Corrigendum: How can the collaborative participation of regulators, whistleblowers, and parties effectively promote rumor management in public health emergencies?

**DOI:** 10.3389/fpubh.2024.1399905

**Published:** 2024-05-21

**Authors:** Yalin Wang, Liping Qi, Shaoshuo Cai

**Affiliations:** ^1^Guangzhou Huashang College, Guangzhou, China; ^2^School of Humanities, University of Chinese Academy of Sciences, Beijing, China; ^3^School of Journalism and Communication, Hunan Normal University, Changsha, China

**Keywords:** public health emergencies, rumor management, collaborative governance, social sustainability, evolutionary games

In the published article, there were some errors within Equations (1)–(6) in Section 3.1, “*Analysis of replication dynamics*.” Specifically:

[(1) In Equations (1) and (2), the term “*Ū*_1_” should be corrected to “*U*_1_”

(2) Equations (3) and (5) redundantly replicate Equation (1), which is not accurate.

(3) Additionally, in Equation (4), “*Ū*_2_” should be modified to “*U*_2_,” and, in Equation (6), “*Ū*_3_” should be adjusted to “*U*_3_”].

A correction has been made to Model analysis, *3.1 Analysis of replication dynamic*. The Equations (1)–(6) previously stated:

“[


(1) $$\{\begin{array}{l} U_{11}=y\left(\left(-C_{o}-gHk\right)\left(1-z\right)+\left(-C_{o}-gHk\right)z\right)+\\ \left(1-y\right)\left(\left(-C_{o}-gHk+mR\right)\left(1-z\right)+\left(-C_{o}-gHk-An+\left(m+n\right)R\right)z\right)\\ U_{12}=y\left(-gkT\left(1-z\right)-gkTz\right)+\left(1-y\right)\left(-gkT\left(1-z\right)+\left(nR-gkT\right)z\right)\\ {\bar{U}}_{1}=xU_{11}+\left(1-x\right)U_{11} \end{array}$$

(2) $$\begin{array}{l} F\left(x\right)=\frac{dx}{dt}=x\left(U_{11}-{Ū}_{1}\right)=\left(-1+x\right)x\left(C_{o}+gk\left(H-T\right)+\left(-1+y\right)\left(mR-Anz\right)\right) \end{array}$$

(3) $$\{\begin{array}{l} U_{11}=y\left(\left(-C_{o}-gHk\right)\left(1-z\right)+\left(-C_{o}-gHk\right)z\right)+\\ \left(1-y\right)\left(\left(-C_{o}-gHk+mR\right)\left(1-z\right)+\left(-C_{o}-gHk-An+\left(m+n\right)R\right)z\right)\\ U_{12}=y\left(-gkT\left(1-z\right)-gkTz\right)+\left(1-y\right)\left(-gkT\left(1-z\right)+\left(nR-gkT\right)z\right)\\ {\bar{U}}_{1}=xU_{11}+\left(1-x\right)U_{11} \end{array}$$

(4) $$\begin{array}{l} F\left(y\right)=\frac{dy}{dt}=y\left(U_{21}-{\bar{U}}_{2}\right)\\ \;=-\left(-1+y\right)y\left(-Dk+Bgk+mRx+nRz\right) \end{array}$$

(5) $$\{\begin{array}{l} U_{11}=y\left(\left(-C_{o}-gHk\right)\left(1-z\right)+\left(-C_{o}-gHk\right)z\right)+\\ \left(1-y\right)(\left(-C_{o}-gHk+mR\right)\left(1-z\right)\\ +\left(-C_{o}-gHk-An+\left(m+n\right)R\right)z)\\ U_{12}=y\left(-gkT\left(1-z\right)-gkTz\right)+\left(1-y\right)(-gkT\left(1-z\right)\\ +\left(nR-gkT\right)z)\\ {\bar{U}}_{1}=xU_{11}+\left(1-x\right)U_{11} \end{array}$$

(6) $$\begin{array}{l} F\left(z\right)=\frac{dz}{dt}=z\left(U_{31}-{\bar{U}}_{3}\right)\\ \;=\left(-1+z\right)z\left(E-gkU+Agnx\left(-1+y\right)\right) \end{array}$$

]”

The corrected Equations (1)–(6) appear below:

“[


(1) $$\{\begin{array}{l} U_{11}=y\left(\left(-C_{o}-gHk\right)\left(1-z\right)+\left(-C_{o}-gHk\right)z\right)+\\ \left(1-y\right)(\left(-C_{o}-gHk+mR\right)\left(1-z\right)\\ +\left(-C_{o}-gHk-An+\left(m+n\right)R\right)z)\\ U_{12}=y\left(-gkT\left(1-z\right)-gkTz\right)+\left(1-y\right)(-gkT\left(1-z\right)\\ +\left(nR-gkT\right)z)\\ U_{1}=xU_{11}+\left(1-x\right)U_{12} \end{array}$$

(2) $$\begin{array}{l} F\left(x\right)=\frac{dx}{dt}=x\left(U_{11}-U_{1}\right)=\left(-1+x\right)x(C_{o}+gk\left(H-T\right)\\ \;+\left(-1+y\right)\left(mR-Anz\right)) \end{array}$$

(3) $$\{\begin{array}{l} U_{21}=\left(1-x\right)\left(-Dk\left(1-z\right)-Dkz\right)+x\left(-Dk\left(1-z\right)-Dkz\right)\\ U_{22}=x\left(\left(-Bgk-mR\right)\left(1-z\right)+\left(-Bgk+\left(-m-n\right)R\right)z\right)+\\ \left(1-x\right)\left(-Bgk\left(1-z\right)+\left(-Bgk-nR\right)z\right)\\ U_{2}=yU_{21}+\left(1-y\right)U_{22} \end{array}$$

(4) $$\begin{array}{l} F\left(y\right)=\frac{dy}{dt}=y\left(U_{21}-U_{2}\right)\\ \;=-\left(-1+y\right)y\left(-Dk+Bgk+mRx+nRz\right) \end{array}$$

(5) $$\{\begin{array}{l} U_{31}=\left(\left(-E+gkU\right)\left(1-x\right)+\left(-E+Agn+gkU\right)x\right)\left(1-y\right)+\\ \left(\left(-E+gkU\right)\left(1-x\right)+\left(-E+gkU\right)x\right)y\\ U_{32}=0\\ U_{3}=zU_{31}+\left(1-z\right)U_{32} \end{array}$$

(6) $$\begin{array}{l} F\left(z\right)=\frac{dz}{dt}=z\left(U_{31}-U_{3}\right)\\ \;=\left(-1+z\right)z\left(E-gkU+Agnx\left(-1+y\right)\right) \end{array}$$

]”

The authors apologize for this error and state that this does not change the scientific conclusions of the article in any way. The original article has been updated.

